# A mindfulness-based intervention improves perceived stress and mindfulness in university nursing students: a quasi-experimental study

**DOI:** 10.1038/s41598-024-64183-5

**Published:** 2024-06-08

**Authors:** Yi-Ling Liu, Chao-Hsien Lee, Li-Min Wu

**Affiliations:** 1 School of Nursing, Kaohsiung Medical University, No. 100, Shin-Chuan 1st Road, Sanmin Dist, Kaohsiung, 807378 Taiwan (R.O.C.); 2 Department of Nursing, Meiho University, Pingtung, Taiwan (R.O.C.); 3 Department of Medical Research, Kaohsiung Medical University Hospital, Kaohsiung, Taiwan; 4 Department of Social Work, Meiho University, Pingtung, Taiwan

**Keywords:** Mindfulness, Perceived stress, Nursing student, Intervention, Psychology, Health care

## Abstract

University nursing students have been shown to experience psychological stress. A mindfulness-based intervention (MBI) may be a helpful tool for stress management. The aim of this study was to examine the effects of a MBI on improving mindfulness and reducing perceived stress in nursing students. A quasi-experimental study was conducted between July 2021 and February 2022. The intervention group participated in an 8-week mindfulness awareness course, which included 50 min of training and practice in mindfulness meditation techniques each week. Over the same 8 weeks, the control group watched a 50-min film each week. The mindful attention awareness scale (MAAS) and perceived stress scale (PSS) were administered before the intervention, intervention completion, and 2 and 6 months after the intervention. Data were analyzed using t test and generalized estimating equation. Overall, that the MBI showed a substantial effect on felt stress in comparison to the control group. When compared to the control group, the MBI showed a substantial impact on trait mindfulness. The MBI was beneficial for nursing students and could be considered a viable approach in nursing education to enhance mental wellbeing. It could be an effective method of relieving stress in a high-stress population.

## Introduction

Mindfulness is an approach to the experience of everyday life that involves the nonjudgmental bringing of attention and awareness to the present moment^1^. It provides individuals with insight and knowledge about their present experiences, including their feelings, thoughts, physical states, consciousness, and environments, while encouraging openness, curiosity, and acceptance^2,3^. Mindfulness can reduce negative thoughts and those about the past and future, and is thought to relieve stress and anxiety and to improve individuals’ resilience^3–5^. The mindfulness-based intervention (MBI) created by Kabat-Zinn^6^ is an 8-week evidence-based program that aims to develop mindfulness, reduce stress, and, overall, improve mental functioning and health^2,5,7–9^. MBIs have been shown to effectively address a wide range of conditions and outcomes, including anxiety, depression, stress, disordered eating, chronic pain, quality of life, and psychological and emotional distress, in diverse populations^10–12^. Loucks et al.^13^ confirmed that MB-College establishes a groundwork of mindfulness abilities, including meditation, yoga, self-awareness, attention control, and emotion regulation, and offers recordings of varying lengths: 10, 20, 30, and 45 min. Students are encouraged to choose the duration that best suits them each day. Growing evidence indicates that MBIs help nurses to reduce stress, and they have been applied in nursing education^14,15^. However, evidence regarding their effectiveness for nursing students is scarce^16^.

Academic stress is the main stress type experienced by nursing students^17^. Due to its potential to affect academic achievement, stress has been recognized as a crucial psycho-social element in the educational process^18^. Students who participate in MBIs report experiencing less stress, more mindfulness, and/or greater empathy^19^. In a review, Chiodelli et al.^20^ confirmed that MBIs were beneficial to undergraduate students overall, findings on the effect of session length (45–120 min) and intervention duration (3–20 weeks) were not consistent. MBIs may be beneficial for nursing students, who according to the transactional analysis theory need to appraise how to manage their stress and emotions when confronting stressful events^21,22^. These students have limited opportunities to cope with various situations during their practice^23^, and they need to continually enrich their knowledge and experience over time to help them reduce stress^23,24^. Developed approach known as Mindfulness-Based Transactional Analysis (MBTA) integrates TA principles with encouraging mindful adult thinking, the authors describe the integration of mindfulness and transactional analysis theory into Mindfulness-Based Transactional Analysis (MBTA). This is an 8-week psychoeducational program that invites participants to cultivate full presence in everyday life, focusing on developing mindfulness capacity^25,26^. In a review, MBIs Transactional Analysis (TA) is applied in drug addiction, where craving beliefs are alleviated through a mindfulness-based TA intervention model. The efficacy of mindfulness-based transactional analysis group therapy in reducing craving beliefs among inpatients at the Indian Noida De-Addiction Center has been demonstrated^27^. The development team needs essential areas of knowledge to create effective Mindfulness-Based Programs tailored to particular populations or contexts. This suggests that mindfulness influences three aspects of self-regulation: attention control, self-awareness, and emotion regulation. Based on MBIs, MBSR, or MB-College, customization and responsive adjustments are continually made according to individual, group, and environmental needs. Teachers are constantly formulating and reformulating content to support participant learning, fine-tuning their teaching to ensure inclusivity and support the needs of individual participants and the entire group (e.g., The Mindfulness-Based Intervention (MBIs) is an evidence-based program lasting for 8 weeks, MBSR suggests the body scan is a 45-min exercise in which attention is directed to any areas of the body while lying down; MB-College offers recordings of varying lengths: 10, 20, 30, and 45 min. Students are encouraged to choose the duration that best suits them each day^13,22,28^. Therefore, nursing students in this study conform to other research suggesting that engaging in 45 min of mindfulness meditation followed by 5 min of physical recovery can achieve overall effectiveness.

Generally, nursing students have higher levels of stress than do other college students, due primarily to the highly demanding and rigorous nature of their professional educational programs^23,29–31^. In addition, nursing students entering clinical practice face major challenges and pressures, such as the need to bridge the gap between theory and practice, the feeling of being underprepared and fear of making mistakes; the need to deal with death and dying and to witness pain and suffering; the need to communicate well with clinical staff, teachers, and patients; and the unfamiliarity of the hospital environment^24,32,33^. The turnover rate for newly graduated nursing staff is high due to the long-term pressure experienced by those in the nursing profession^33^. However, burnout is indeed becoming increasingly prevalent among professional healthcare students. Burnout syndrome can manifest in any environment characterized by chronic stress, including academic settings^34^. Furthermore, university studies in fields like Nursing or Psychology, which are related to the healthcare system, present additional risk factors for developing burnout syndrome, such as exposure to human suffering and the responsibility for others’ health^35^. The incidence of burnout syndrome is significant among university students, with main stressors identified including concerns about exam performance, adapting to the university environment, study demands, and uncertainty about the future (González Ramírez and Landero Hernández 2007). Comprehending the occurrence and impacts of burnout within graduate healthcare programs enables faculty and administration to design curricula and offer information to students, helping them understand, identify, and seize opportunities to mitigate burnout, thereby fostering the development of enduring, high-quality clinicians^36^. This study was to test the efficacy of a mindfulness-based training program on reducing stress and increasing positive thinking of nursing students. Similar to their U.S. counterparts, nursing students in Taiwan experience stress and mindfulness, which may affect their academic and clinical performance. Therefore, this study was designed to examine the effects of the program on stress and mindfulness of nursing students in Taiwan.

## Methods

A longitudinal quasi‐experimental design, repeated-measures study was performed with a 6-month follow-up period to investigate the effects of an 8-week mindfulness awareness course on the mindfulness and perceived stress of nursing students before their clinical practice. Cluster randomization was employed to assign two classes to the experimental and control groups, mitigating the risk of contamination, as participants within clusters are likely to share experiences and similar learning environments.

### Participants and setting

This study was approved by the Institutional Review Board (or Ethics Committee) of the Antai Medical Care Cooperation Antai-Tian-Sheng Memorial Hospital (protocol code, 21-045-B, and date of approval, 15 July 2021). This study followed the principles described in the Helsinki Declaration. All research activities were carried out in conformity with the applicable rules and regulations. The text includes a statement stating that all participants or their legal guardians provided informed consent. The recruitment period was July 2021–February 2022. Participants were third-year nursing students enrolled in the 5-year nursing program at a university, southern Taiwan. Eligible students spoke and read Mandarin or Taiwanese clearly; those who had taken a similar mindfulness course previously, regularly practiced mindfulness, or had completed their nursing practicum were excluded. All participants, and legal representatives of those aged < 20 years, provided written informed consent to study participation. The students received no credit for their participation.

The G*Power software (Ver. 3.1) was utilized to determine that the sample size was estimated to be 41 participants per group, with a power of 0.8, alpha of 0.05, and effect size of 0.25. This estimation was based on a repeated-measures between-factor analysis of variance of data collected from four assessment timepoints^37^.

### Intervention

The intervention was based on the current MBI standard^6^ and the transactional theory concept that stress derives from relationships between people and their environments^21^. Participants in the experimental group took an 8-week mindfulness awareness course that included training and practice in mindfulness meditation techniques for 50 min/week. Those in the control group watched a 50-min film once a week for 8 weeks. An overview of the intervention content is provided in Table 1. The participants were taught by the first author who was trained to use mindfulness meditation techniques such as the body scan, a 30-min exercise in which the individual, while sitting in a relaxed position with the eyes closed, sequentially directs his or her attention to specific areas of the body and carefully observes how each area feels. Participants were instructed in meditation, with the direction of their attention to the sensations of breathing, tension, and relaxation. They also learned an exercise in which they focused on the feeling of rolling two wooden balls in the palm of the dominant hand for 15 min, repeating the movement dozens of times. They were taught to practice mindfulness skills in daily activities such as walking, standing, and eating. The participants were asked to practice the skills and techniques for 45 min/day, with a final 5-min period to return to the perception of all bodily sensations. After the completion of the course, the researchers used the LINE application^38^ to remind the participants in the intervention group to practice for at least one hour per week and during periods of low mood.

**Table 1:** The mindfulness-based training program.

Week	Subject	Goals	Mindfulness exercise
1	Build connections	Course introduction Let others know yourself	Learn to observe Participate meaningfully
2	Interpersonal interaction	Care about yourself and others Promote positive emotions Promote optimism	Discover good things and make yourself feel good
3	Skillful responses	Skillful practice Choose skillful responses	Respond consciously Make the determination to get out of trouble
4	Body scan	Relief techniques Awareness practice	Practice tension and relaxation
5	Focus on awareness	Mindfulness-focused practice	Feel the movement of wooden balls between the palm and fingers
6	Positive emotions	Connect with positive emotions Connect with positive people	Use mindfulness techniques when encountering difficulties Practice gratitude and caring for those around you
7	External resources	Strengthen positive traits and learn to find resources Keep positive resources	Use life skills Enhance learning effect
8	Integration exercises	Integrate each other’s growth Give back to and support each other	Integrate course takeaways Bless each other

### Measurements

#### Mindful attention awareness scale (MAAS)

The 15-item MAAS, developed by Brown and Ryan^39^, is used to determine the degree of individuals’ mindfulness. Respondents score items about inattentive states on a Likert scale ranging from 1 (almost always) to 6 (almost never). Participants were asked how frequently you currently had experienced. Higher scores reflect greater mindfulness^39^. The Cronbach’s α value for the original MAAS was 0.81^39^, and it was 0.83 in our study.

#### Perceived stress scale (PSS)

The 14-item PSS was developed by Cohen et al.^40^. It was used to measure the level of unpredictable, uncontrollable and overloaded stress experienced by the respondents in the past month. Responses are structured by a 5-point Likert scale (0 = never, 4 = always). Total scores range from 0 to 56, with higher scores reflecting more perceived stress^40^. The concurrent validity of the original scale was tested and Cronbach’s α values were 0.84–0.86^40^, and it was 0.79 in our study.

### Data collection

Classes were randomly assigned to experimental and control groups due to the possibility of contamination. whereby participants within clusters are likely to have shared experiences and similar learning environments^41^. Before the start of the intervention (T0), the researcher explained the study purpose and procedures to the students and asked them to provide consent and to take 20–30 min to complete a questionnaire (the content of which was explained). At T0, items soliciting data on students’ gender (female or male), age, religious beliefs (yes or no), monthly family income (20,000–59,999 or > 60,000 NTD), and personality (e.g., extroverted, easy-going, conscientious, gentle, open-minded), and their parents’ teaching style (democratic, authoritative, permissive, uninvolved, or inconsistent) were included. The Mindful Attention Awareness Scale (MAAS) and Perceived Stress Scale (PSS) were administered at T0, upon completion of the intervention (T1), and at 2 (T2) and 6 (T3) months after the intervention.

### Data analysis

The data were analyzed using SPSS 24.0 (IBM Corporation, Armonk, NY, USA). Categorical variables are reported as frequency and percentages and continuous variables are reported as mean and standard deviations. Differences between groups at the same timepoint were assessed using the t test. Considering time effects, a generalized estimating equation (GEE) was used to examine differences on MASS and PSS between groups at different time points^42–44^.

### Ethical committee approval

This study was approved by the Institutional Review Board (or Ethics Committee) of the Antai Medical Care Cooperation Antai-Tian-Sheng Memorial Hospital (protocol code, 21-045-B, and date of approval, 15 July 2021). Additionally, we also registered our study on the ISRCTN registry with study registration number ISRCTN13821133 on May 24, 2024.

## Results

An 8-week course on mindfulness-awareness, comprising weekly 50-min sessions of instruction and practice in mindfulness meditation techniques, was administered to participants in the experimental group. Meanwhile, participants in the control group watched a 50-min movie once a week for 8 weeks during the same period. Each participant was evaluated four times using the MASS and PSS scales: before the intervention (T0), immediately following the intervention (T1), 2 months after the intervention (T2), and 6 months after the intervention (T3). By comparing score differences at the four time points and changes in scores over time between the control and intervention groups, we assessed the intervention’s impact on MASS and PSS measures.

**Figure 1:**
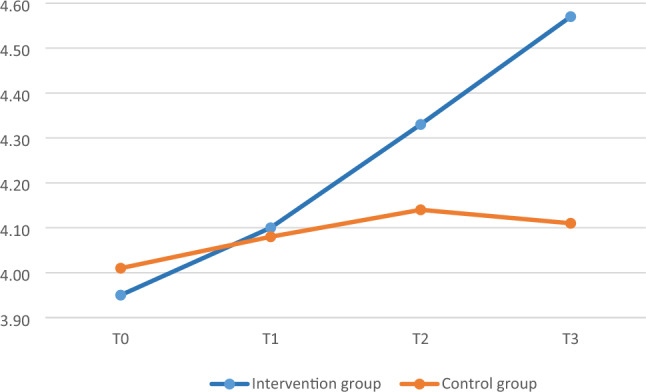
The trend of the MAAS scores between the intervention and control groups at T0, T1, T2, and T3.

**Figure 2:**
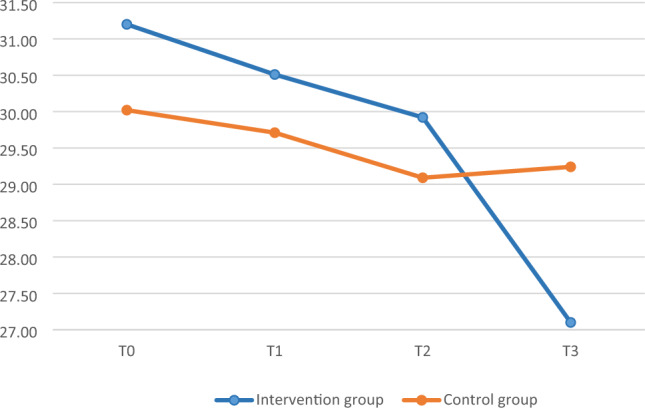
The trend of the PSS scores between the intervention and control groups at T0, T1, T2, and T3.

Mean MASS and PSS scores were plotted to illustrate group differences at the four assessment times. Baseline scores (T0) were similar between groups for all measurements. Over time, participants in the intervention group showed an increase in mean MASS scores (Fig. 1) and a decrease in mean PSS scores (Fig. 2). No adverse events or unexpected issues occurred during the intervention.

### Participant characteristics

All of the 94 students (10 males, 84 females) whose eligibility was evaluated participated in this study and completed all procedures. The 49 participants, with mean age 18.22 (SD = 0.42) years old, were allocated to the intervention group, while the 45 participants, with mean age 18.27 (SD = 0.45) years old, were allocated to the control group. Their baseline characteristics are summarized in Table 2. More than 80% of the participants reported having religious beliefs and 34 students reported family incomes > $60,000 NTD. The participant characteristics did not differ between groups. No adverse event or unexpected issue occurred during the intervention.

**Table 2:** Participant characteristics (*n* = 94).

Characteristic	Control		Intervention		*χ*^2^	*p*
	*n*	%	*n*	%		
Gender						
Female	43	95.6	41	87.7	3.48	0.094
Male	2	4.4	8	16.3		
Religious beliefs						
No	6	13.3	7	14.3	0.20	0.894
Yes	39	86.7	42	85.7		
Monthly family income						
20,000–59,999 NTD	30	66.7	30	61.2	0.30	0.583
≥ 60,000 NTD	15	33.3	19	38.8		
Personality traits						
Extroverted	16	35.6	12	24.5	4.77	0.445
Easy-going	22	48.9	23	46.9		
Conscientious	2	4.4	4	8.2		
Gentle	2	4.4	4	8.2		
Open-minded	2	4.4	6	12.2		
Other	1	4.4	0	0		
Parents’ teaching style						
Democratic	32	71.1	39	79.5	5.36	0.252
Authoritative	5	11.1	3	6.1		
Permissive	0	0	2	4.1		
Uninvolved	0	0	1	2		
Inconsistent	8	17.8	4	8.2		

*MAAS* mindful attention awareness scale, *PSS* perceived stress scale.

### Within-group changes in MAAS and PSS scores

The mean MAAS score increased and the PSS score decreased over time in the intervention group (Figs. 1, 2). The MAAS score was significantly higher at T2 and T3 than at T0 (*t* = 3.51, *p* = 0.001 and *t* = 4.17, *p* < 0.001, respectively); no significant difference was observed between T1 and T0 (Table 3). The PSS score was significantly lower at T3 than at T0 (*t* =  − 3.52, *p* = 0.001), but did not differ from baseline at T1 or T2 (Table 3). No significant change in the MAAS or PSS score was observed in the control group (Table 3).

**Table 3:** Differences in MAAS and PSS scores.

	Control group				Intervention group			
	Mean difference	SD	*t*	*p*	Mean difference	SD	*t*	*p*
MAAS								
T1–T0	0.07	0.55	0.89	0.380	0.14	0.52	1.93	0.060
T2–T0	0.13	0.66	1.35	0.185	0.38	0.76	3.51*	0.001
T3–T0	0.11	0.62	1.14	0.261	0.61	1.03	4.17***	< 0.001
PSS								
T1–T0	− 0.31	5.85	− 0.36	0.723	− 0.69	4.28	− 1.13	0.262
T2–T0	− 0.93	6.06	− 1.03	0.307	− 1.29	5.81	− 1.55	0.128
T3–T0	− 0.78	6.63	− 0.79	0.435	− 4.10	8.16	− 3.52**	0.001

*MAAS* mindful attention awareness scale, *PSS* perceived stress scale, *SD* standard deviation, *T0* before intervention, *T1* immediately after intervention, *T2* 2 months after intervention, *T3* 6 months after intervention.

*P < 0.05, **P < 0.01, ***P < 0.001.

### Between-group differences in MAAS and PSS scores at different timepoints

GEE modeling revealed between-group differences in the MAAS and PSS scores over time (Table 4). Group and time had no main effect on either score, but significant (group × time) interaction was found for the difference at T3 in the MAAS (Wald* χ*^2^ = 8.71, *p* = 0.003) and PSS (Wald *χ*^2^ = 4.84, *p* = 0.028) scores, reflecting improvements in the intervention group between T0 and T3. The difference in the MAAS and PSS scores between T0 and T3 were 0.51 and 3.32 points greater, respectively, in the intervention group than in the control group.

**Table 4:** Effects on MAAS and PSS scores.

Measure	β	SE	95% CI	Wald *χ*^2^	*p*
MAAS					
Intercept	4.01	0.10	[3.81, 4.21]	1643.49***	< 0.001
Group (intervention vs. control)	− 0.05	0.15	[− 0.34, 0.24]	0.13	0.724
Time (T1 vs. T0)	0.07	0.08	[− 0.09, 0.23]	0.82	0.365
Time (T2 vs. T0)	0.13	0.10	[− 0.07, 0.33]	1.89	0.170
Time (T3 vs. T0)	0.11	0.10	[− 0.09, 0.31]	1.35	0.245
Group × time (T1)	0.07	0.11	[− 0.15, 0.29]	0.41	0.522
Group × time (T2)	0.25	0.15	[− 0.04, 0.54]	2.85	0.092
Group × time (T3)	0.51	0.17	[0.18, 0.84]	8.71**	0.003
PSS					
Intercept	30.02	0.89	[28.28, 31.76]	1144.34***	< 0.001
Group (intervention vs. control)	1.18	1.34	[− 1.45, 3.81]	0.77	0.379
Time (T1 vs. T0)	− 0.31	0.86	[− 2.00, 1.38]	0.13	0.718
Time (T2 vs. T0)	− 0.93	0.89	[− 2.67, 0.81]	1.09	0.296
Time (T3 vs. T0)	− 0.78	0.98	[− 2.70, 1.14]	0.63	0.426
Group × time (T1)	− 0.38	1.05	[− 2.44, 1.68]	0.13	0.716
Group × time (T2)	− 0.35	1.21	[− 2.72, 2.02]	0.08	0.772
Group × time (T3)	− 3.32	1.51	[− 6.28, − 0.36]	4.84*	0.028

*MAAS* mindful attention awareness scale, *PSS* perceived stress scale, *SD* standard deviation, *T0* before intervention, *T1* immediately after intervention, *T2* 2 months after intervention, *T3* 6 months after intervention.

*P < 0.05, **P < 0.01, ***P < 0.001.

### Correlations between the difference scores of MAAS and PSS at each stage compared to T0 in intervention group

We observed correlations between the increase in specific items of MAAS and the decrease in PSS at different time points (Table 5). The correlation coefficients between MAAS (T1 vs T0) and PSS (T1 vs T0), PSS (T2 vs T0), and PSS (T3 vs T0) were − 0.651 (p < 0.001), − 0.572 (p < 0.001), and − 0.551 (p < 0.001), respectively. For MAAS (T2 vs T0) and PSS (T2 vs T0), and PSS (T3 vs T0), the correlation coefficients were − 0.673 (p < 0.001) and − 0.648 (p < 0.001), respectively. Finally, the correlation coefficient between MAAS (T3 vs T0) and PSS (T3 vs T0) was − 0.759 (p < 0.001).

**Table 5:** Correlations between the difference scores of MAAS and PSS at each stage compared to T0 in intervention group.

Difference scores at each stage compared to T0	PSS		
	T1 vs T0	T2 vs T0	T3 vs T0
MAAS			
T1 vs T0	− 0.651***	− 0.572***	− 0.551***
T2 vs T0		− 0.673***	− 0.648***
T3 vs T0			− 0.759***

***P < 0.001.

## Discussion

Our findings supported our hypothesis that participants in the experimental group significantly increase mindfulness and decrease perceived stress over 6 months follow-up. Significantly differences were found on mindfulness and perceived stress for participants between two groups after 6 months follow-up. Our intervention was an 8-week mindfulness-based intervention and combined the LINE software to remind participants self-practice every day. Song and Lindquist^22^ MBSR program were the standard elements of yoga, sitting, walking, breath-work, body scan, and eating meditation.

One of the unique aspects of this study was that we scheduled an 8-week, 50-min weekly mindfulness meditation skill instruction and practice course. The course can be replicated in other ethnic groups and clinical units. The main technique is to use two mindfulness meditation techniques: The first is to conduct a 30-min body scanning technique practice through the instructor, allowing participants to scan the body during meditation and carefully observe every aspect of the body. Feel in each area, and focus on the awareness of breathing, walking, tension, and relaxation. The second is to use a 15-min wooden ball focus training, allowing participants to focus on the feeling of rolling the ball on the palm, teaching the body to focus on mindfulness, and guiding participants to apply it to daily activities, such as walking, standing and eating. In addition, the line software is another feature that continuously remind participants to practice daily life for the 6 months follow-up.

### Mindfulness

The mindfulness significantly increased over time in the experimental group. Although no significantly improved after intervention immediately, it significantly increased at 2 months and 6 months after the intervention exception in intervention group. Additionally, significant differences of mindfulness were found between two groups after participants had received an 8 weeks intervention after 6 months. Our findings were confirmed as a 8 weeks mindfulness-based intervention was shown to be significantly beneficial to the nursing students. Our findings were similar to Horrillo Álvarez et al.^45^, but were not consistent with previous studies^16,22^. In Horrillo Álvarez et al.^45^ study, after 47 voluntary participants aged 20–60 received 8 weeks (total 8 h) of mindfulness training, there was no significant difference in mindfulness between the two groups after 8 weeks. However, the immediate effects were found in other two studies^16,22^. The possible reasons might be: (1) higher does and intensity training. In Song and Lindquist^22^ study, they provided 2 h per week and continued for 8 weeks (total 16 h). In Cheli et al.^16^ pre-post study design, the intervention was composed of five 3-hs sessions and 4.5 h (total 19.5 h). The dose provided by Cheli et al.^16^ is 2.44 times than ours (the total dose of this study is 8 h), and the interventions are all completed within 6 weeks. In Martínez-Rubio et al.^36^ study, they offered abbreviated programs (under 8 weeks), shorter sessions (90 min), brief practices (15 min or less), and prioritized training through informal practice. Furthermore, there was a significant emphasis on the effectiveness of mindfulness practice, particularly informal practice, as the primary means for personal transformation. In this program, participants were allocated 9 h for formal at-home practice, with sessions lasting 15 min each. Overall, the program is distinguished by its emphasis on short practice sessions and underscores the significance of informal practice in integrating mindfulness into daily routines. The current standard of mindfulness-based interventions, designed by Kabat-Zinn^6^, involves 26 h of session time consisting of eight weekly classes of 2.5 h each and an all-day 6-h class during sixth week^46^. According to the systematic review: the duration of mindfulness-based interventions is between 3 and 20 weeks, and the time of each section is diverse (40–120 min)^20^. More researches are needed to clarity this phenomenon. (2) Participants come from self-selection process that causes a biased sample to enhance the treatment effect in above studies. Thus, future intervention research should consider these issues to improve the outcomes.

In a review, Berghoff et al.^47^ confirmed that assessed the relationships among adherence, meditation practice time, and psychiatric symptoms following two 2-week mindfulness meditation interventions: one prescribing 10 min of daily meditation and another prescribing 20 min of daily meditation. The findings revealed no significant group difference in total days of meditation or overall meditation time. However, stress levels decreased and mindfulness increased over the 2-week period for both groups. Despite similar adherence rates, participants in the 20-min group reported greater increases in self-compassion compared to those in the 10-min group. Ma et al.^48^ programs like MBSR, MBCT, and their derivatives typically require a significant commitment to program sessions (usually eight weekly sessions of 2 h each) and home practice (45 min of daily practice), which may not resonate with university students and often leads to high dropout rates. This contradicts our research. The possible reasons might be: (1) Due to the younger age and higher compliance of our nursing students, we chose to follow Kabat-Zinn^6^ is an 8-week evidence-based program, which entails 50 min of training and practice. (2) During the long-term follow-up, there was no loss of our case count. Researchers used the LINE application to remind students to practice daily and record their progress.

### Perceived stress

The results of this study show that the perceived pressure reached a significant difference at the 6th month (T3) after the intervention between two groups, indicating that the significant differences were found after participants practicing for 6 months. The perceived pressure did not decrease immediately after the intervention that were similar to the previous studies (Horrillo Alvarez et al. 2022)^49^, but not found in Kang et al.^50^, and Burger and Lockhart^51^. Our study did not show an immediate effect after the intervention. The reasons for this may include: (1) varied training time, such as the 17.5 h reported in the study by Kang et al.^50^ or 10 min of online videos per day for more than 4 weeks of practice in the study by Burger and Lockhart^51^. (2) According to the transactional model of stress and coping^21^, individuals, after assessing their stress levels, may make cognitive or behavioral adjustments to reduce the stress. In this study, the mindfulness scores in the experimental group increased over time and showed a significant increase at 2 months after the intervention. Additionally, perceived stress also exhibited a significant difference between the two groups at 6 months post-intervention. It is possible that participants gradually increased their mindfulness, leading to a reduction in perceived stress. Our findings revealed that mindfulness practice needs to be continued over a long period to achieve the expected benefits. This study confirmed that mindfulness practice can enhance self-awareness to help individuals adapt to stress^4^, and reduces stress among nursing students^52,53^. Therefore, our intervention, an 8-h training course before nursing practice, can be utilized to help nursing students continuously practice mindfulness skills on their own, enhancing mindfulness and reducing stress.

Therefore, regardless of the length or intensity of mindfulness practice, each study has its limitations. In the future, we must address and overcome these research challenges. The goal is to equip students with effective self-healing and stress-reducing techniques, enabling them to handle various clinical pressures. Our aim is to strengthen and empower students to become more resilient.

## Conclusions

This study showed that an 8-h mindfulness-based training program for nursing students increased mindfulness and reduced perceived stress at 6 months after program completion. Thus, such training should be promoted for nursing students, with the recognition that continual practice over time is needed to fully integrate mindfulness skills. Our findings show that mindfulness practice needs to be continued for 6 months period to achieve the expected benefits, and confirm that mindfulness practice enhances self-awareness to adapt to stress and reduces stress among nursing students. Thus, our intervention can be feasibly implemented before nursing students enter their practice, followed by the continuous practice of the skills learned to enhance mindfulness and reduce stress.

In the future, more nursing students can benefit from mindfulness skills exercises, allowing them to feel more at ease during clinical practice. This approach may also help reduce the turnover rate among clinical nursing staff.

## Limitations

This study was limited by the inclusion only of participants from the nursing program of a single technological university, which reduces the generalizability of the findings. Additionally, to accurately ascertain the effects of MBIs, studies conducted with larger and more diverse participant samples are needed. Despite these limitations, however, we provide a structured 8-h mindfulness-based course for nursing students that had measurable beneficial effects.

## Data Availability

It was not publicly available for the dataset during the current study since they contain potentially identifiable information for each participant; however, it is available from the corresponding author upon reasonable request.
